# What affects the bone in our HIV-positive patients?

**DOI:** 10.1186/1758-2652-13-S4-O41

**Published:** 2010-11-08

**Authors:** CA Fux

**Affiliations:** 1 Universitätsklinik fur Infektiologie, Inselspital, Bern, Switzerland

## 

Osteopenia and osteoporosis are frequent in HIV infected patients, with a prevalence of 66% and 15% reported in a metaanalysis. In an aging population, low bone mineral density (BMD) will translate in fracture-related morbidity and mortality over time. As a matter of fact, increased fracture rates in HIV positive patients have recently been reported, particularly if older than 40-50 years. The etiology of low BMD is multifactorial. HIV independent risk factors may thereby overweight HIV dependent and treatment related factors. Low BMI, malnutrition, vitamin D deficiency, substance abuse, hypogonadism, physical inactivity or osteotoxic medication (e.g. steroids), chronic liver or kidney disease are overrepresented in many HIV positive populations. HIV positivity correlates with increased bone turnover. Viral replication results in continuous cytokine production that directly and indirectly (through RANKL) activates osteoclasts. On the other hand, SMART and several other studies have correlated cART initiation with accelerated bone loss irrespective of the regimen used. Remarkably, this effect stabilized within the first year of treatment and might thus be related to IRIS. Most, but not all, associations of bone loss with protease inhibitor treatment disappeared after correction for HIV-independent risk factors for low BMD, in particular low BMI. Upon Tenofovir initiation, a similar pattern has been observed even in switch studies with suppressed viremia. Again, these findings normalized within a year. Based on a concomitant increase in serum alkaline phosphatase and PTH levels, osteomalacia secondary to drug-related renal phosphate wasting has been postulated, but lacks strong evidence. Still, monitoring of phosphatemia and the correction of vitamin D deficiency has been suggested for Tenofovir-treated patients.

Taken together, HIV positive patients carry a relevant risk for low-impact fractures. This merits a systematic assessment of risk factors for low BMD and falls. Given that a prevalent vertebral fracture indicates an equal risk for a subsequent fracture as documented osteoporosis, particular attention should be paid to identify subclinical fractures. For patients aged over 40, FRAX® can be used to identify patients qualifying for bone density measurement (DXA) or biphosphonate treatment according to national guidelines. HIV may thereby be considered as a secondary cause of osteoporosis.

